# Gene polymorphisms of inflammatory factors in liver cirrhosis

**DOI:** 10.3389/fgene.2023.1140427

**Published:** 2023-04-10

**Authors:** Sailan Xiao, Xiongfeng Pan, Xun Huang, Yamin Liu, Shi Wu Wen, Aizhong Liu

**Affiliations:** ^1^ Department of Epidemiology and Health Statistics, Xiangya School of Public Health, Central South University, Changsha, China; ^2^ Clinical Epidemiology Program, Ottawa Hospital Research Institute, Ottawa, ON, Canada; ^3^ Department of Obstetrics and Gynaecology, University of Ottawa Faculty of Medicine, Ottawa, ON, Canada; ^4^ School of Epidemiology and Public Health, University of Ottawa Faculty of Medicine, Ottawa, ON, Canada; ^5^ Hunan Provincial Key Laboratory of Clinical Epidemiology, Xiangya School of Public Health, Central South University, Changsha, China

**Keywords:** inflammatory factors, gene polymorphism, liver cirrhosis, systematic review, meta-analysis

## Abstract

**Introduction:** Studies on the association between gene polymorphisms of various inflammatory factors and liver cirrhosis have been inconsistent. The purpose of this study was to comprehensively summarize the available evidence on the association between gene polymorphisms of inflammatory factors and liver cirrhosis through a systematic review.

**Methods:** We searched databases of PubMed, EMBASE, Web of Science, and the Cochrane Library for relevant articles published from building databases to 25 September 2022. A systematic review and meta‐analysis were performed to investigate the association between gene polymorphisms of various inflammatory factors and liver cirrhosis. Odds ratios (OR) and 95% confidence intervals (CI) were used to assess the strength of association.

**Results:** A total of 43 articles were included in the systematic review and of them, 22 articles were included in the meta‐analysis. The gene polymorphisms of IL-10–1082 GA + AA vs. GG (OR = 1.43, 95% CI = 1.12–1.83), IL-10–1082 AA vs. GG (OR = 2.03, 95% CI = 1.36–3.02), IL-18 -137 GG vs. CC (OR = 3.84, 95% CI = 1.29–11.40), TGF-β1 -509 T vs. C (OR = 2.52, 95% CI = 1.42–4.48), and IFN-γ +874 T vs. A (OR = 1.98, 95% CI = 1.32–2.98) were associated with liver cirrhosis significantly and no association was observed in other gene polymorphisms included in the meta‐analysis. The review of inflammatory factors gene polymorphisms that were only reported by a single study indicated 19 gene polymorphisms were risk factors and 4 gene polymorphisms were protective factors for liver cirrhosis, while the association between other 27 gene polymorphisms and liver cirrhosis were not statistically significant.

**Discussion:** This study suggests that IL-10 -1082G/A, IL-18 -137G/C, TGF-β1 -509T/C, and IFN-γ +874T/A were potentially associated with the risk of liver cirrhosis susceptibility. These findings may provide comprehensive evidence for genetic susceptibility and immunogenetic pathology of liver cirrhosis.

## 1 Introduction

Liver cirrhosis (LC) is a common chronic progressive liver disease, characterized by diffuse fibrosis, pseudo lobule formation, and eventually to portal hypertension, liver failure, or hepatocellular carcinoma (HCC) (Parola and Pinzani, 2019; Gines et al., 2021). It is reported that more than 1.32 million deaths are attributable to liver cirrhosis worldwide, accounting for 2.4% of global total deaths (G. B. D. C. Collaborators, 2020), and liver cirrhosis is the 11th most common cause of death and the third leading cause of death in people aged 45–64 years (Asrani et al., 2019). Nevertheless, there is no effective treatment for liver cirrhosis. Although antifibrotic or pro-regenerative drug therapies for cirrhosis have been approved, they have been in final stage of clinical trials and the effect has not been definitively determined (Fallowfield et al., 2021). A detailed understanding of cirrhosis pathogenesis may contribute to the development of new therapies.

The main causes of liver cirrhosis were chronic infection of hepatitis B virus (HBV) or hepatitis C virus (HCV), alcoholic liver disease (ALD), and non-alcoholic fatty liver disease (NAFLD). Other causes include primary biliary cirrhosis (PBC), primary sclerosing cholangitis (PSC), autoimmune hepatitis (AIH), and genetic and metabolic diseases, such as Wilson’s disease and hemochromatosis (Gines et al., 2021). These pathogenic processes involve a sequence of inflammation, during which various inflammatory factors play a key role (Bernsmeier et al., 2020). They can induce the immune system to secrete inflammatory factors or directly mediate liver cell damage. In the process of regulatory immune responses, the recruitment of both pro- and anti-inflammatory cells such as monocytes and macrophages amplify the response through the production of other inflammatory factors, which increase the stimulus of hepatic stellate cells (HSC) by activating proinflammatory cells (Altamirano-Barrera et al., 2017).

Furthermore, inflammatory factors themselves are manipulated by polymorphisms in their genes. Single nucleotide polymorphisms (SNPs) are the commonest form of genetic variants in human being and they may affect the level of inflammatory factors production and the intensity of their response. There is increasing evidence on the association between individual genetic polymorphisms of inflammatory factors and liver diseases, including chronic hepatitis, liver cirrhosis, and HCC (Aleagha et al., 2020; Wei et al., 2021). The genes mainly include Interleukin genes (IL), interferon-γ genes (IFN-γ), transforming growth factor-β genes (TGF-β), and tumor necrosis factor-α genes (TNF-α). Among them, IL-10, TGF-β and TNF-α have been studied the most. Previous studies have reported various inflammatory factors gene polymorphisms in liver cirrhosis, but the results were not consistent. Identification of genetic factors related to susceptibility to liver cirrhosis would help to elucidate the complex process of the disease and improve the scientific basis for therapeutics or preventive interventions.

So far, several common inflammatory factors gene polymorphisms in liver cirrhosis have been investigated by meta-analysis (Chen et al., 2011; Guo et al., 2015; Guo et al., 2018; Zheng et al., 2021; Liu and Yang, 2022). However, to our knowledge, there has not been a systematic review and meta-analysis that has comprehensively assessed the association of inflammatory factors gene polymorphisms with liver cirrhosis. Therefore, the purpose of this systematic review and meta‐analysis was to comprehensively summarize the available evidence on the association between various different inflammatory factors gene polymorphisms and liver cirrhosis.

## 2 Methods

### 2.1 Search strategy

This systematic review and meta‐analysis were performed according to the Preferred Re‐porting Items for Systematic Reviews and Meta‐Analyses (PRISMA) guidelines (Moher et al., 2009). We systematically searched PubMed, EMBASE, Web of Science, and the Cochrane Library databases for relevant articles published from building databases to 25 September 2022. The search strategy included all possible combinations of keywords related to liver cirrhosis, inflammatory factors, and their associated outcomes. The search keywords were as follows: Liver Cirrhosis OR Hepatic Cirrhosis OR Cirrhosis, Hepatic OR Cirrhosis, Liver OR Fibrosis, Liver OR Liver Fibrosis AND Interleukin OR Cytokine OR Interferon OR Lymphocyte OR Macrophage OR Microglia OR Tumor Necrosis Factor OR C-Reactive Protein OR Transforming growth factor OR IFN* OR IL* OR CRP* OR TGF* OR TNF* OR CSF* OR GF* OR MCP* OR CCL* OR CXCL* OR Inflammatory factor* OR Pro-inflammatory cytokine* OR colony stimulating factor OR Chemokine OR Inflammatory cytokine.

### 2.2 Selection criteria

Studies that met all of the following criteria were included: 1) the study design was case-control, cohort or cross-sectional; 2) the study investigated the association between inflammatory factors gene polymorphisms and liver cirrhosis; 3) the study established liver cirrhosis group and control group without liver cirrhosis; 4) diagnoses for liver cirrhosis were reported; 5) genotype frequencies were in line with Hardy–Weinberg equilibrium (HWE). Studies that met any of the following criteria were excluded from the study: 1) duplicated studies retrieved from various databases; 2) reviews, meta-analyses, conference papers, comments or case reports; 3) animal or *in vitro* cell experiments; 4) no full text; 5) no complete odds ratio (OR) and 95% confidence interval (95% CI).

### 2.3 Data extraction

Endnote 20 was used to manage articles and three investigators assessed articles and extracted relevant data from eligible articles independently, then a customized data extraction Excel spreadsheet was used to extract the data for this study. Differences among the three independent reviewers were resolved by another reviewer. The following data were extracted from the eligible studies: 1) the last name of the first author and year of publication; 2) the SNP of various inflammatory factors; 3) study design and sample size; 4) OR and 95% CI; 5) country where the research was conducted and race of participants; 6) type of case and control and diagnostic criteria; 7) material of sample, storage temperature, and genotyping method; 8) patients characteristics such as Mean age, Male gender, Body Mass Index (BMI), Aspartate aminotransferase (AST), Alanine transaminase (ALT) and γ-Glutamyl transpeptidase (GGT). Finally, the Newcastle-Ottawa Quality Assessment Scale (NOS) was used to assess the quality of eligible studies.

### 2.4 Statistical analysis

RevMan5.3 software was used for meta-analysis. OR and 95% CI were used to assess the strength of associations. Using Q test and I^2^ statistic to test the degree of heterogeneity. If *p* > 0.05 and I^2^ <50%, the results are homogeneous, and the fixed effect model is used for combined analysis; otherwise, the random effect model is used for combined analysis. Because the number of included studies of single SNP was less than 10, no formal assessment of publication bias was performed. Sensitivity analysis were conducted when I^2^ ≥ 50%. The sensitivity analysis adopted the method of eliminating individual studies item by item to explore the source of heterogeneity.

## 3 Results

### 3.1 Study selection

**FIGURE 1:**
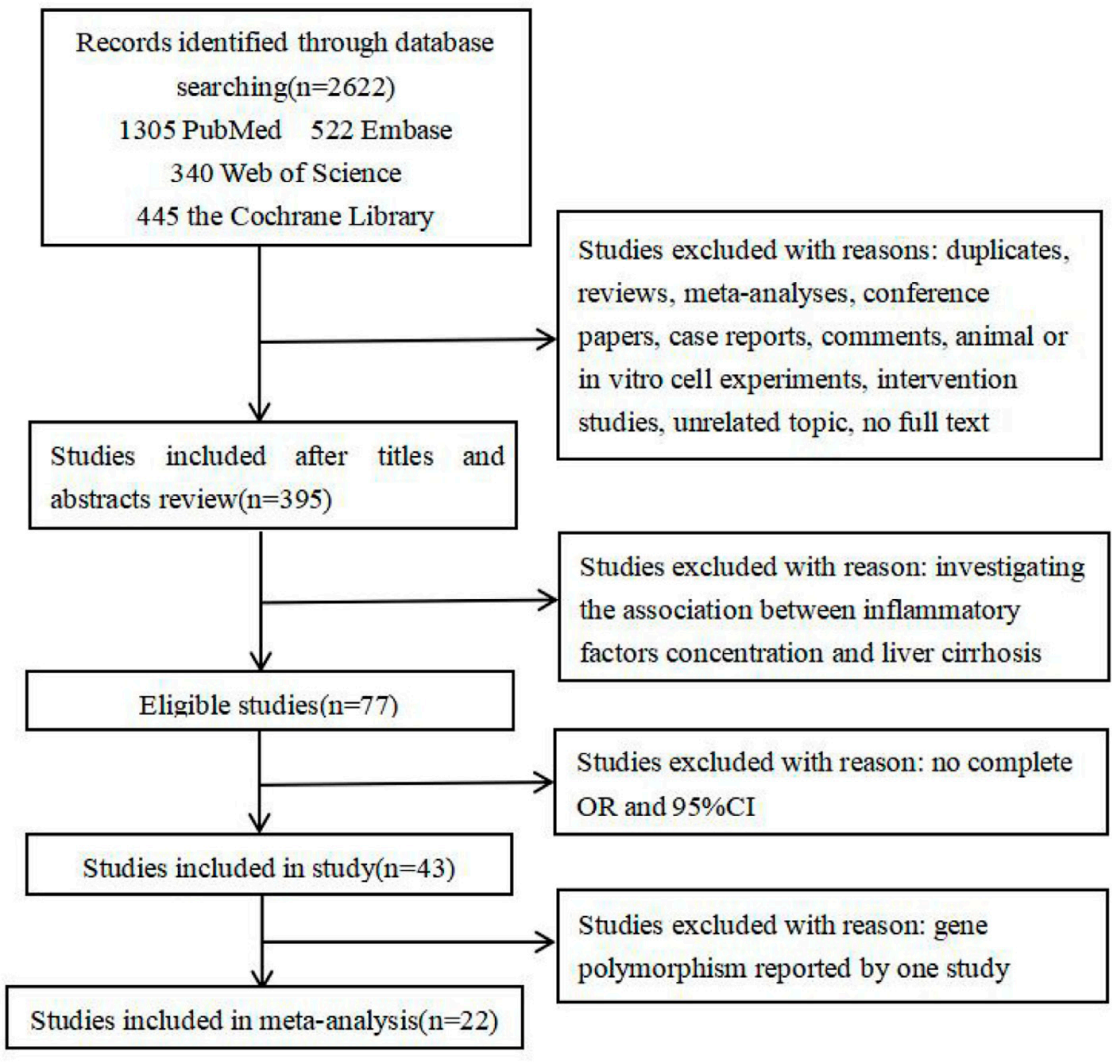
The flowchart of study selection.

The search strategy retrieved a total of 2,622 studies, of which 1,305 were from PubMed, 522 from Embase, 340 from Web of Science, and 445 from Cochrane Library. The assessment of full texts of the 395 articles found that 318 articles examined the association between inflammatory factors concentration and liver cirrhosis and 34 provided incomplete OR and 95% CI, and were hence excluded, leaving 43 articles for the systematic review. Among them, 22 were included in the meta-analysis (Figure 1).

### 3.2 Study characteristics

Characteristics of the 22 included articles in the meta-analysis are presented in Table 1. These studies were published between 2003 and 2022 and 7 studies were conducted in China, 5 in Egypt, 2 in Germany, 2 in Tunisian, 2 in Italy, 1 in Mexico, 1 in Turkey, 1 in Spain, and 1 in Thailand. Among 2,500 cases, 516 were HCV-induced liver cirrhosis, 803 were HBV-induced, 105 were alcohol-induced, 200 were Opisthorchis viverrini infection-induced, and 876 were mixed. Among 2,984 controls, 2,789 were healthy controls and 195 were chronic hepatitis C infection patients. PCR was used in 4 studies, PCR-RFLP in 12 studies, AS-PCR in 3 studies, ARMS-PCR in 2 studies, and TaqMan in 2 studies. Seven inflammatory factors and 12 SNPs were identified. In addition, the quality scores of study quality assessment varied between 6 and 8, with 8 studies classified as moderate quality and 14 as high quality. The assessment sheet is shown in Supplementary Appendix S1.

**TABLE 1:** Characteristics of included studies.

Study	Country	Case		Control		Genotyping method	SNP	Quality score
		Type	No.	Type	No.			
Armendáriz-Borunda 2008 Armendáriz-Borunda et al. (2008)	Mexico	HCV-LC	13	HC	30	PCR	TGF-β1 Arg25Pro	6
Bahgat 2015 Bahgat et al. (2015)	Egypt	HCV-LC	50	CHC	25	AS-PCR	IFN-γ +874/IL-10 -1082	6
Bahr 2003 Bahr et al. (2003)	Germany	HCV-LC	52	HC	200	PCR	IL-1β −511/TNF-α −308	7
Bouzgarrou 2008 Bouzgarrou et al. (2008)	Tunisian	HCV-LC	47	CHC	34	PCR-RFLP	IL-18 -137	6
Bouzgarrou 2009 Bouzgarrou et al. (2009)	Tunisian	HCV-LC	58	CHC	42	AS-PCR	IFN-γ +874/IL-10 -1082	6
Cao 2016 Cao et al. (2016)	China	HBV-LC	241	HC	254	PCR-RFLP	IL-10 -592/IL-10 -1082	7
Dai 2017 Dai et al. (2017)	China	HBV-LC	250	HC	250	PCR	IL-18 -137	8
Fabris 2011 Fabris et al. (2011)	Italy	Mixed-LC	138	HC	344	PCR-RFLP	IL-28 rs12979860	7
Falleti 2008 Falleti et al. (2008)	Italy	Mixed-LC	188	HC	140	PCR	TGF-β1 Arg25Pro	6
Liu 2015 Liu et al. (2015)	China	Mixed-LC	192	HC	192	PCR-RFLP	IL-10 -592/IL-10 -819/IL-10 -1082	7
Lu 2015 Lu et al. (2015)	China	HBV-LC	86	HC	160	PCR-RFLP	IL-18 -137/IL-18 -607	8
Mohy 2014 Mohy and Fouad (2014)	Egypt	Mixed-LC	40	HC	40	PCR-RFLP	TGF-β1 -509	7
Nomair 2021 Nomair et al. (2021)	Egypt	HCV-LC	70	CHC	20	TaqMan	TGF-β1 Arg25Pro	6
Öksüz 2022 Öksüz et al. (2022)	Turkey	HCV-LC	24	CHC	24	TaqMan	IL-28 rs12979860	6
Pastor 2005 Pastor et al. (2005)	Spain	Alcohol-LC	65	HC	84	PCR	TNF-α −238	7
Petrásek 2009 Petrásek et al. (2009)	Germany	HBV-LC	100	HC	180	PCR-RFLP	IL-1β −511	8
Radwan 2012 Radwan et al. (2012)	Egypt	HCV-LC	152	HC	160	PCR-RFLP	TGF-β1 -509	7
Sheneef 2017 Sheneef et al. (2017)	Egypt	HCV-LC	50	CHC	50	PCR-RFLP, ARMS-PCR	IFN-γ +874/IL-10 -592/IL-10 -1082	6
Sun 2015 Sun et al. (2015)	China	HBV-LC	126	HC	173	PCR-RFLP	IFN-γ +874	7
Surapaitoon 2017 Surapaitoon et al. (2017)	Thailand	O viverrini-LC	200	HC	200	AS-PCR	IFN-γ +874/IL-1β −511	7
Yang 2014 Yang et al. (2014)	China	Alcohol-LC	40	HC	64	PCR-RFLP, ARMS-PCR	IL-10 -819/IL-10 -1082/IL-1β −511	7
Yao 2015 Yao et al. (2015)	China	Mixed-LC	318	HC	318	PCR-RFLP	IL-10 -592/IL-10 -819/IL-10 -1082	7

HBV/HCV, hepatitis B/C virus infection; CHB/CHC, chronic hepatitis B/C; LC, liver cirrhosis; HC, healthy control; Mixed, mixed etiology; O viverrini, Opisthorchis viverrini infection; PCR-RFLP, polymerase chain reaction followed by restriction fragment length polymorphism; ARMS, amplification refractory mutation system; AS, allele-specific.

### 3.3 Association between gene polymorphisms of inflammatory factors and liver cirrhosis

**FIGURE 2:**
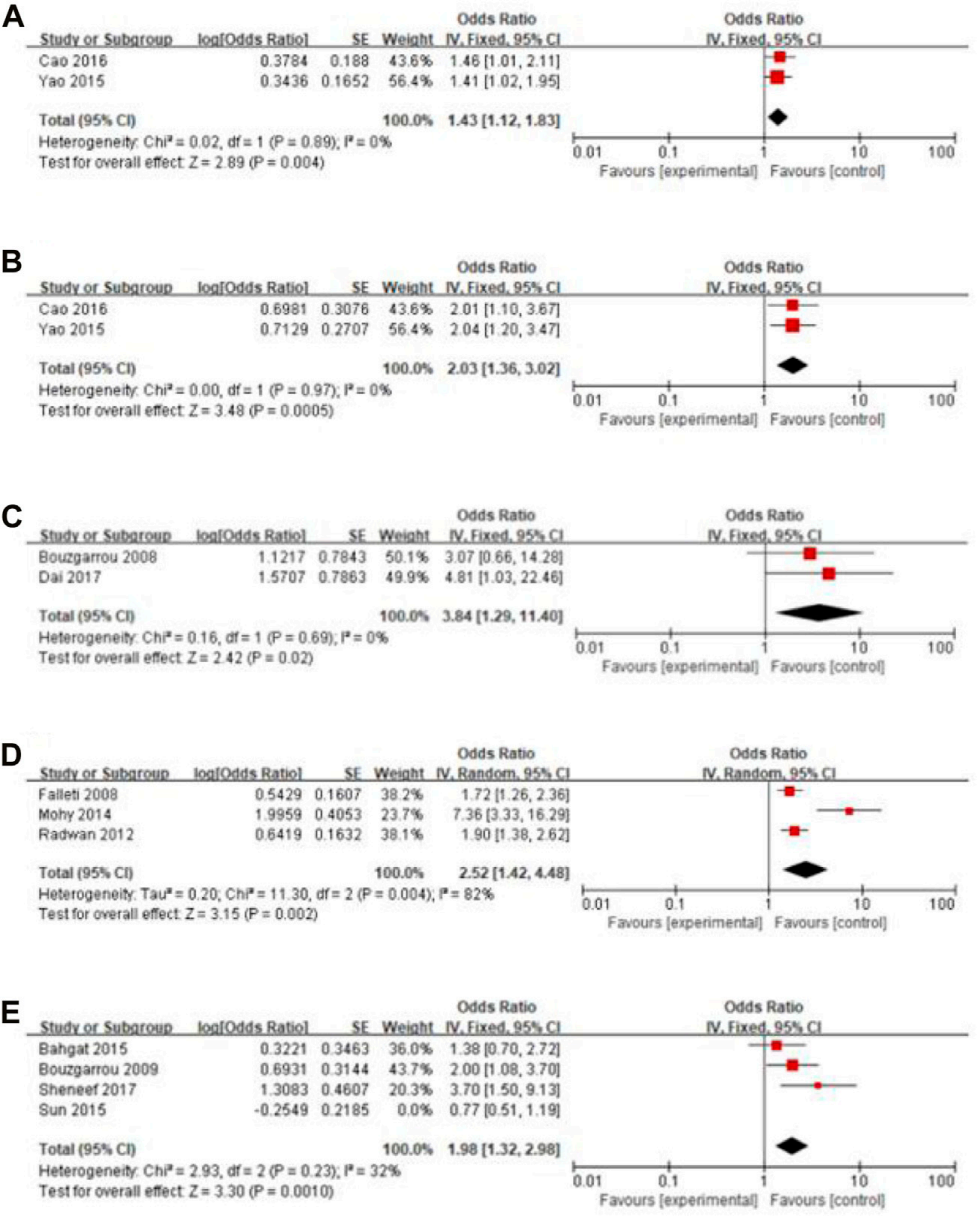
Forest plot of the significant results. **(A)** IL-10–1082 GA + AA vs. GG; **(B)** IL-10–1082 AA vs. GG; **(C)** IL-18 -137 GG vs. CC; **(D)** TGF-β1 -509 T vs. C; **(E)** IFN-γ +874 T vs. A.

Gene polymorphisms of IL-10–1082 GA + AA vs. GG (OR = 1.43, 95% CI = 1.12–1.83), IL-10–1082 AA vs. GG (OR = 2.03, 95% CI = 1.36–3.02), IL-18 -137 GG vs. CC (OR = 3.84, 95% CI = 1.29–11.40), and TGF-β1 -509 T vs. C (OR = 2.52, 95% CI = 1.42–4.48) were significantly associated with liver cirrhosis. The sensitivity analysis showed that in the study of IFN-γ +874 T vs. A, the heterogeneity of other studies was significantly reduced and the result was reversed (OR = 1.98, 95% CI = 1.32–2.98) after excluding the article by Sun et al. (2015). Figure 2 displays the forest plot of these results. No association was observed in other SNPs included in the meta‐analysis (Table 2) and the forest plots are shown in Supplementary Appendix S2.

**TABLE 2:** Association between gene polymorphisms of inflammatory factors and liver cirrhosis.

	No. of studies	OR (95% CI)	*p*	I^2^ (%)	*p* for heterogeneity	Effect model
IFN-γ +874T/A						
TT vs. AA	3	1.36 (0.49, 3.80)	0.560	52	0.120	random
TA vs. AA	2	0.89 (0.55, 1.44)	0.620	0	0.820	fixed
TT + TA vs. AA	3	1.30 (0.60, 2.80)	0.690	61	0.210	random
TT vs. TA + AA	2	1.07 (0.31, 3.74)	0.920	66	0.009	random
T vs. A	4	1.57 (0.82, 2.99)	0.170	76	0.005	random
IL-10 -1082G/A						
GG vs. AA	3	0.82 (0.34, 2.01)	0.670	44	0.090	fixed
GA vs. AA	4	1.03 (0.72, 1.46)	0.880	48	0.120	fixed
GG + GA vs. AA	2	0.63 (0.31, 1.30)	0.210	0	0.700	fixed
AA vs. GG + GA	2	1.14 (0.45, 2.89)	0.780	69	0.070	random
G vs. A	4	1.06 (0.65, 1.73)	0.820	56	0.080	random
IL-10–592C/A						
CC vs. AA	3	1.32 (0.95, 1.83)	0.100	0	0.970	fixed
AC vs. AA	3	1.16 (0.93, 1.45)	0.190	0	0.820	fixed
AC + CC vs. AA	2	1.16 (0.90, 1.49)	0.260	0	0.870	fixed
IL-10–819C/T						
TT vs. CC	2	1.69 (0.73, 3.92)	0.220	47	0.170	fixed
CT vs. CC	2	1.92 (0.42, 8.66)	0.400	57	0.130	random
IL-18–607A/C						
CC vs. AA	2	1.35 (0.42, 4.34)	0.620	54	0.140	random
CA vs. AA	2	1.64 (0.56, 4.86)	0.370	55	0.140	random
IL-18 -137G/C						
GC vs. CC	2	1.55 (0.53, 4.52)	0.430	51	0.150	random
CG + GG vs. CC	2	1.34 (0.89, 2.02)	0.160	40	0.200	fixed
IL-1β −511C/T						
CC vs. TT	2	0.81 (0.41, 1.59)	0.540	2	0.310	fixed
CT vs. TT	2	0.90 (0.45, 1.77)	0.750	0	0.500	fixed
T vs. C	2	1.08 (0.83, 1.40)	0.560	15	0.280	fixed
IL-28 rs12979860C/T						
TT vs. CT + CC	2	3.90 (0.27, 55.75)	0.320	70	0.070	random
TGF-β1 Arg25ProC/G						
G vs. C	3	2.28 (0.82, 6.37)	0.002	82	0.004	random
TNF-α −308G/A						
GA vs. GG	2	1.48 (0.42, 5.26)	0.540	77	0.040	random
A vs. G	3	1.40 (0.72, 2.75)	0.320	84	0.002	random
TNF-α −238G/A						
GA vs. GG + AA	2	2.18 (0.56, 8.54)	0.260	80	0.003	random

**FIGURE 3:**
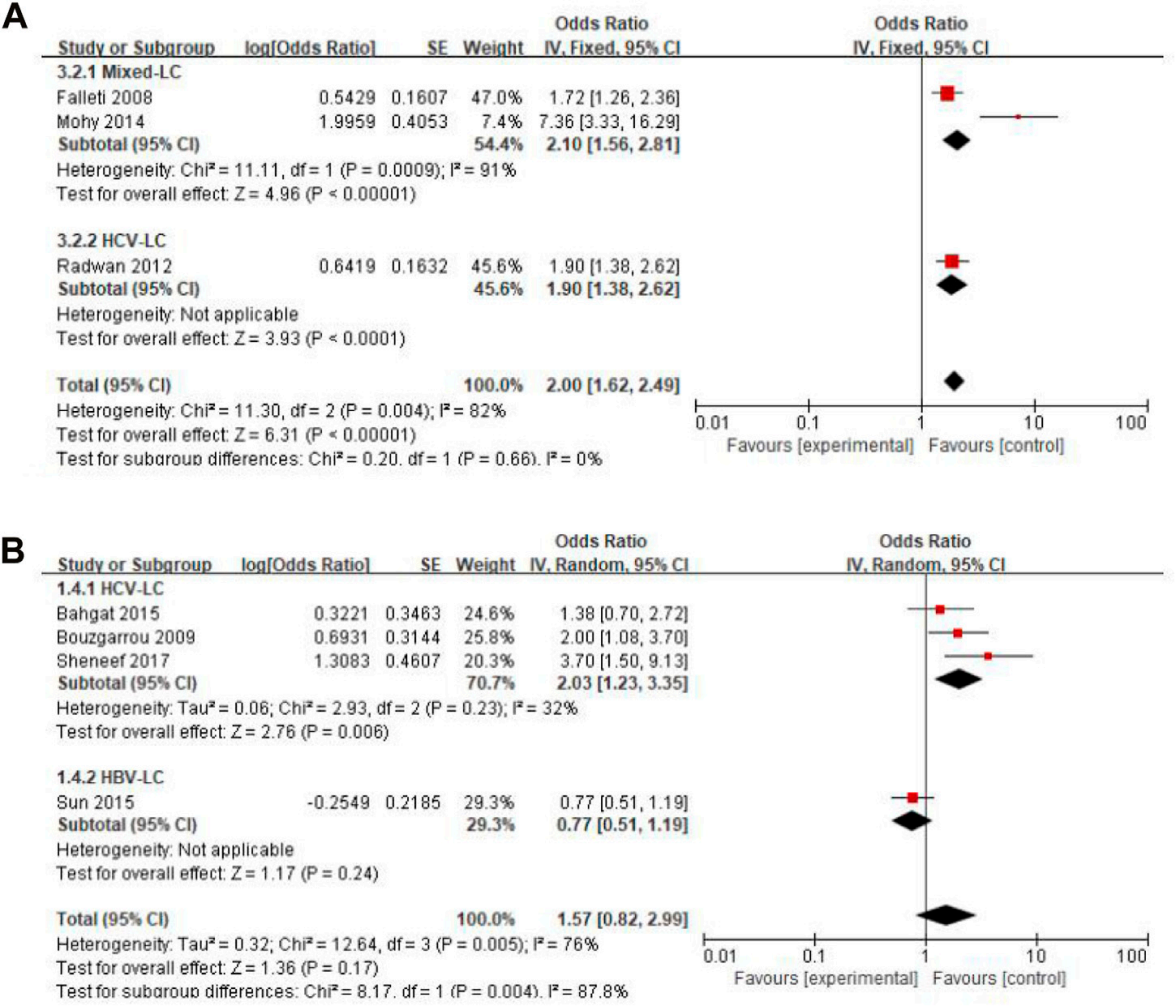
Forest plot of subgroup analyses by different etiologies of liver cirrhosis. **(A)** TGF-β1 -509 T vs. C; **(B)** IFN-γ +874 T vs. A.

Considering that liver cirrhosis has different etiologies, we performed subgroup analyses by classification of study subjects into HBV-LC, HCV-LC, Alcohol-LC, O viverrini-LC, and Mixed-LC. These analyses showed that there was no difference in gene polymorphisms of TGF-β1 -509 T vs. C between Mixed-LC and HCV-LC groups (I^2^ = 0%, *p* = 0.660), while there was a significant difference of IFN-γ + 874 T vs. A between HCV-LC and HBV-LC groups (I^2^ = 88%, *p* = 0.004) and heterogeneity was reduced in the subgroup of the HCV-LC group (Figure 3). Subgroup analyses for IL-10–1082 GA + AA vs. GG, IL-10–1082 AA vs. GG, and IL-18 -137 GG vs. CC were not conducted for each etiology of cirrhosis reported by only one study. Forest plots of subgroup analyses of other insignificant SNPs are shown in Supplementary Appendix S3.

### 3.4 Association between gene polymorphisms of other related inflammatory factors and liver cirrhosis

In addition to the above inflammatory factors included in the meta-analysis, some inflammatory factors gene polymorphisms that were only reported by a single study. As part of the systematic review, their findings on gene polymorphisms are summarized in Supplementary Appendix S4. The results indicated that gene polymorphisms of CXCL-1 (rs4074G/A), IFR5(rs13242262A/T, rs10488630A/G), IL-13 (rs1800925A/T), IL-17 (rs4711998A/G), IL-1α(rs3783553del/ins), IL-22 (rs1026788A/G, rs1179249A/C, rs2227472A/G, rs2227485C/T, rs2227491A/G), IL-33 (rs1048274A/G, rs10975519T/C, rs4742170T/C), IL-6 (rs10499563T/C, rs2069837A/G), MIF(-173G/C), TNF-α(-857C/T, −863C/A) were risk factors for liver cirrhosis and gene polymorphisms of IFN-γ(+2109A/G, IL-2 (-330T/G), IL-6 (rs1474347A/C), IL-8 (-251A/T) were protective factors for liver cirrhosis. No significant association of gene polymorphisms of IL-13 (rs20541A/T), IL-17 (rs4711998A/G, rs763780C/T), IL-1α(-889A2A2), IL-1β(-3953A2A2, -31C/T), IL-2 (+114T/G, −384T/G), IL-22 (rs2046068A/C, rs2227473A/G, rs7314777T/C), IL-27 (-964A/G, 2905T/G), IL-28 (rs8099917T/G), IL-4 (-589C/T, −33C/T), IL-6 (rs1800796G/C, rs1800795G/C), IL-8 (+781C/T), IRF3(-925A/G), LT-α(+252A/G), TGF-β1 (-800G/A, Leu10ProC/T), TNFAIP3(rs148314165T/T del, rs200820567T/A, rs2230926G/T), VEGFA (+936C/T) with liver cirrhosis were found.

## 4 Discussion

Only a few people develop cirrhosis after chronic liver injury such as viral infection, alcohol consumption, and metabolic, cholestatic or autoimmune damages, indicating that the modifiers determine the progress of these individuals towards liver cirrhosis. In addition to the recognized progression factors such as age, gender, race and exposure time window or duration, unknown individual genetic factors may affect disease progression. Inflammation is primarily responsible for the pathological progression of liver fibrosis and cirrhosis (Alegre et al., 2017; Koyama and Brenner, 2017). Inflammatory factors are a kind of endogenous polypeptides, produced mainly by immune system cells and are capable of mediating a variety of immune responses. Hence, we performed this systematic review and meta-analysis to explore the association between various inflammatory factors gene polymorphisms and liver cirrhosis. We found that IL-10 (−1082 GA + AA and AA), IL-18 (−137 GG), TGF-β1 (−509 T) and IFN-γ (+874 T) were potentially associated with the risk of liver cirrhosis susceptibility. Subgroup analyses showed that the results of TGF-β1 -509 T vs. C were consistent with the total analysis, indicating that the result was stable and not affected by different etiologies. While the associations of IFN-γ +874 T with liver cirrhosis vs. A and liver cirrhosis were affected by different etiologies, and the results were consistent with the sensitivity analysis.

**FIGURE 4:**
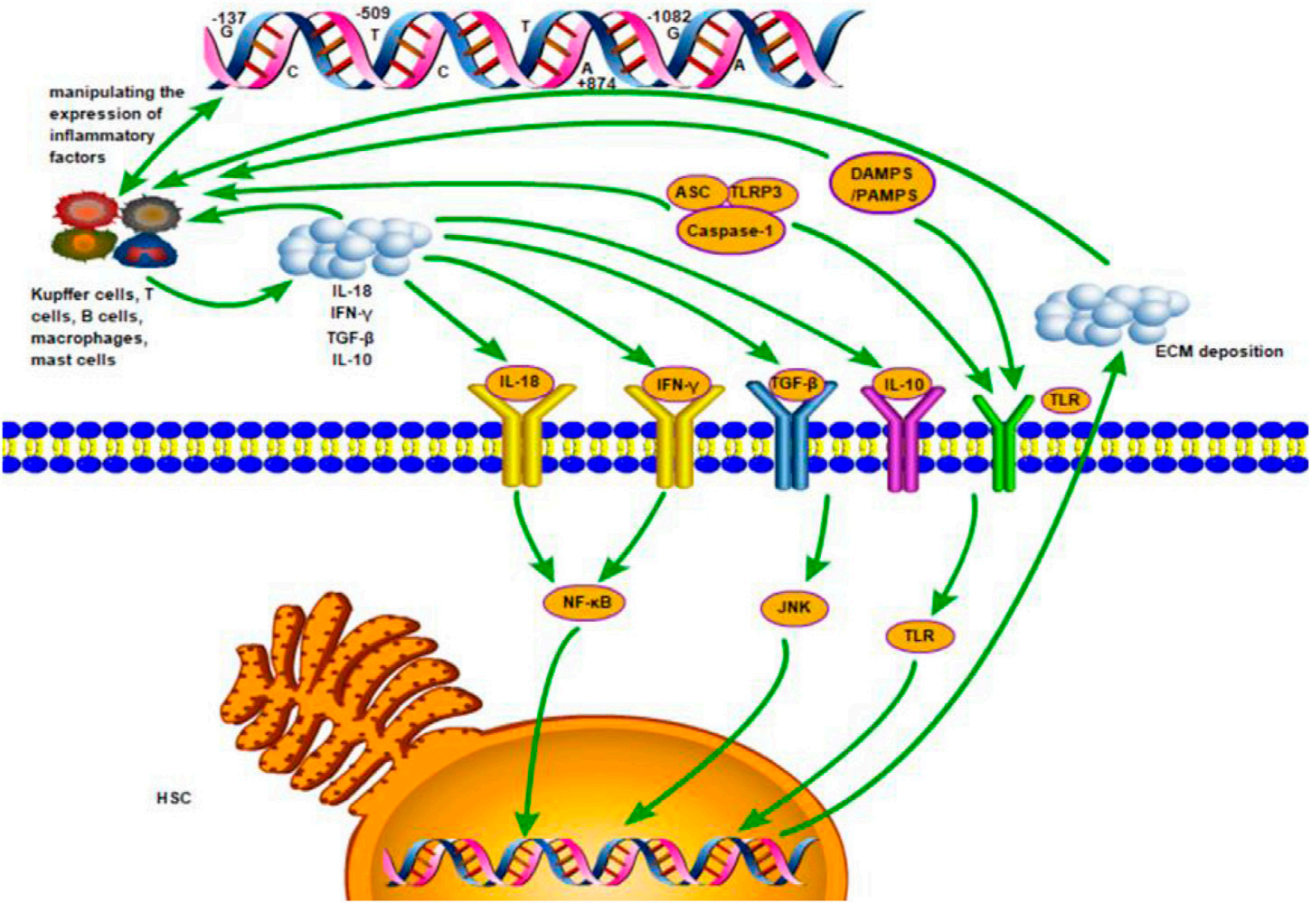
The inflammation mechanism of liver fibrosis mediated by inflammatory factors. Various immune cells (i.e., T cells B cells, macrophages mast cells and specifically Kupffer cells) are recruited in the liver tissue. These immune cells further release more inflammatory cytokines (i.e., IL-10, IL-18, TGF-β1 and IFN-γ), which, together with damage-associated molecular pattern (DAMPS), pathogen-associated molecular patterns (PAMPS) or inflammasomes released by injured or dead liver cells, induce more secretion of inflammatory factors and stimulate HSC by activating the nuclear factor kappa B (NF-kB) pathway, the c-Jun N terminal kinase (JNK) pathway and the Toll-like receptor (TLR) signaling pathway. The active HSC secrete much extracellular matrix (ECM) and cause deposition, so that chronic liver inflammation persists and leads to the formation of liver fibrosis. These processes are controlled by related genes.

HSC is the main receptor cell of inflammatory signal and the main effector cell of promoting fibrosis (Parola and Pinzani, 2019). In the process of inflammation, various immune cells (i.e., T cells B cells, macrophages mast cells and specifically Kupffer cells) are recruited in the liver tissue. These immune cells further release more inflammatory cytokines (i.e., IL-10, IL-18, TGF-β1 and IFN-γ), which, together with damage-associated molecular pattern (DAMPS), pathogen-associated molecular patterns (PAMPS) or inflammasomes released by injured or dead liver cells, can induce more secretion of inflammatory factors and stimulate HSC by activating various inflammatory pathways. The nuclear factor kappa B (NF-kB) pathway (Ramos-Tovar and Muriel, 2020), the c-Jun N terminal kinase (JNK) pathway (Seki et al., 2012) and the Toll-like receptor (TLR) signaling pathway (Leifer and Medvedev, 2016) were well-known pathways. The active HSC secrete much extracellular matrix (ECM) and cause deposition, so that chronic liver inflammation persists and leads to the formation of liver fibrosis. These processes are controlled by related genes (Figure 4). On the other hand, the degeneration and necrosis of liver cells cause liver cell regeneration and connective fiber tissue proliferation. The mechanism of liver cirrhosis is complex and has not been clearly understood yet. These processes may be primarily responsible for the progression of liver cirrhosis.

TGF-β1 has been proven to be the most effective major cytokine to promote liver fibrosis by variable paths (Jeong et al., 2011; Fabregat et al., 2016; Tsuchida and Friedman, 2017). This study suggests that TGF-β1 -509T allele carriers maybe more likely to develop cirrhosis than C carriers. This was supported by the results of a meta-analysis (Guo et al., 2018) although the included literature was different. Our research did not include in other gene loci or comparative models due to the availability of original studies. IL-10 is an effective anti-inflammatory cytokine, mainly produced by Th2 cells, macrophages, and mast cells. IL-10 therapy has been reported to reduce the severity of liver inflammation and fibrosis (Nelson et al., 2000). In the promoter polymorphisms influencing the level of IL-10, the −1082G/A polymorphism is of some importance. This study indicates that IL-10–1082GA + AA and AA genotype carriers are more likely to develop cirrhosis than GG carriers. We included 7 pairs of comparison and no association was observed in other five comparison models. IL-18 plays a role in inflammation through the induction of inflammatory cytokines such as IFN-γ (Yoshimoto et al., 1998). This study indicates that IL-18 -137GG genotype carriers were more likely to develop cirrhosis than CC carriers. We included 3 pairs of comparison and no association was observed in other 2 comparison models. IFN-γ is one of the most important Th1 cytokines. Significant correlation was observed between expression level of IFN-γ mRNA and stage of fibrosis in chronic hepatitis C (Gigi et al., 2008). This study suggests that IFN-γ +874T allele carriers were more likely to develop cirrhosis than A carriers. This is the result of sensitivity analysis, and the sensitivity analysis of the other 4 comparative models also shows no correlation. We found that liver cirrhosis in excluded studies was caused by HBV, while other studies were caused by HCV, which suggested that the cause of liver cirrhosis was the source of heterogeneity. Considering the complex mechanisms of genetic action, further studies are needed to confirm the effects of these polymorphisms on inflammatory factors expression.

Our results showed that there were no significant associations of IL-10 (-592C/A, −819C/T), IL-18 (-607A/C), IL-1β −511C/T, IL-28 rs12979860C/T, TGF-β1(Arg25ProC/G), TNF-α (−308G/A, −238G/A) with liver cirrhosis. One of the reasons for this result may be the limited number of eligible studies. Gene polymorphisms of IL-10, IL-18, TGF-β1 have been confirmed to be related to liver cirrhosis by our study, however, it is accepted that IL-1β, IL-28, especially TNF-α plays an important role in the occurrence and development of liver cirrhosis. More research is needed to explore the relationship between these inflammatory factor gene polymorphisms and liver cirrhosis.

IL-6, a primary immunomodulatory cytokine, has been shown related to liver cirrhosis in a few studies. Zheng et al. (2021) identified three polymorphisms in IL-6 gene and perform a meta-analysis to explore their association with liver cirrhosis. Five polymorphisms (rs1474347, rs10499563, rs2069837, rs1800795, rs1800796) in IL-6 gene were also identified in our systematic review. It worths noting that the polymorphisms (rs1800795, rs1800796) identified simultaneously in both studies had the same results. However, we could not perform a meta-analysis to summarize the association because of the limitation of number of studies. Broader searches may be able to identify more studies on the association between IL-6 gene polymorphisms and liver cirrhosis. Besides, there were other inflammatory factors gene polymorphisms were only reported by single original study. We identified a few genetic polymorphisms this way and more studies are needed to further explore their association with liver cirrhosis.

This study has some limitations that should pay attention to. Firstly, only four databases were searched and some relevant original studies may be missed. Secondly, there were many predisposing factors such as age and race for liver cirrhosis, but we did not carry out further subgroup analysis on factors due to the limited number of relevant studies. Thirdly, we did not conduct a further analysis due to the lack of data on the severity of liver fibrosis, these may influence the precision of the association. Fourthly, only SNP was considered in this review due to the dearth of studies on the combined impact of gene-gene polymorphisms and haplotype gene polymorphisms. As liver cirrhosis is a multi-factors disease, the combined effects of factors like gene-gene and gene-environment should be considered. Moreover, liver cirrhosis and inflammatory diseases of the intestinal tract like inflammatory bowel diseases may share susceptibility gene loci, and expanded search of more databases may unveal different biomarkers.

## 5 Conclusion

Our systematic review and meta-analysis suggest that IL-10 -1082G/A, IL-18 -137G/C, TGF-β1 -509T/C and IFN-γ +874T/A are potentially associated with the risk of liver cirrhosis susceptibility. These findings may provide comprehensive evidence for genetic susceptibility and immunogenetic pathology of liver cirrhosis.

## Data Availability

The original contributions presented in the study are included in the article/Supplementary Material, further inquiries can be directed to the corresponding author.
