# Supplementary material for: Gene polymorphisms of inflammatory factors in liver cirrhosis
Source: Front Genet. 2023 Apr 10;14:1140427. doi: 10.3389/fgene.2023.1140427 (PMC10123281; doi:10.3389/fgene.2023.1140427)
Supplement: Supplementary file 1 [file Table1.DOCX]

Supplementary Material

Gene Polymorphisms of Inflammatory Factors in Liver Cirrhosis: A Systematic Review and Meta‐Analysis

Sailan Xiao, Xiongfeng Pan, Xun Huang, Yamin Liu, Shi Wu Wen, Aizhong Liu

Corresponding Author: Dr. Aizhong Liu

E-mail: lazroy@live.cn

**Contents**

**Appendix 1:** Quality score sheet

**Appendix 2:** Forest plots of the insignificant results

**Appendix 3:** Association between gene polymorphisms of other inflammatory factors and liver cirrhosis

**References**

**Appendix 1:** Quality score sheet

| Study | Selection | | | | Comparability | Exposure | | | Score |
| --- | --- | --- | --- | --- | --- | --- | --- | --- | --- |
|  | a | b | c | d |  | e | f | g |  |
| Armendáriz-Borunda 2008 | 0 | 1 | 1 | 1 | 1 | 1 | 1 | 0 | 6 |
| Bahgat 2015 | 0 | 1 | 0 | 1 | 2 | 1 | 1 | 0 | 6 |
| Bahr 2003 | 1 | 1 | 1 | 1 | 1 | 1 | 1 | 0 | 7 |
| Bouzgarrou 2008 | 1 | 1 | 0 | 1 | 1 | 1 | 1 | 0 | 6 |
| Bouzgarrou 2009 | 1 | 1 | 0 | 1 | 1 | 1 | 1 | 0 | 6 |
| Cao 2016 | 1 | 1 | 1 | 1 | 1 | 1 | 1 | 0 | 7 |
| Dai 2017 | 1 | 1 | 1 | 1 | 2 | 1 | 1 | 0 | 8 |
| Fabris 2011 | 1 | 1 | 1 | 1 | 1 | 1 | 1 | 0 | 7 |
| Falleti 2008 | 0 | 1 | 1 | 1 | 1 | 1 | 1 | 0 | 6 |
| Liu 2015 | 1 | 1 | 1 | 1 | 1 | 1 | 1 | 0 | 7 |
| Lu 2015 | 1 | 1 | 1 | 1 | 2 | 1 | 1 | 0 | 8 |
| Mohy 2014 | 1 | 1 | 1 | 1 | 1 | 1 | 1 | 0 | 7 |
| Nomair 2021 | 1 | 1 | 0 | 1 | 1 | 1 | 1 | 0 | 6 |
| Öksüz 2022 | 1 | 1 | 0 | 1 | 1 | 1 | 1 | 0 | 6 |
| Pastor 2005 | 1 | 1 | 1 | 1 | 1 | 1 | 1 | 0 | 7 |
| Petrásek 2009 | 1 | 1 | 1 | 1 | 2 | 1 | 1 | 0 | 8 |
| Radwan 2012 | 1 | 1 | 1 | 1 | 1 | 1 | 1 | 0 | 7 |
| Sheneef 2017 | 1 | 1 | 0 | 1 | 1 | 1 | 1 | 0 | 6 |
| Sun 2015 | 1 | 1 | 1 | 1 | 1 | 1 | 1 | 0 | 7 |
| Surapaitoon 2017 | 1 | 1 | 1 | 1 | 1 | 1 | 1 | 0 | 7 |
| Yang 2014 | 1 | 1 | 1 | 1 | 1 | 1 | 1 | 0 | 7 |
| Yao 2015 | 1 | 1 | 1 | 1 | 1 | 1 | 1 | 0 | 7 |

a, Is the case definition adequate? b, Representativeness of the cases. c, Selection of controls. d, Definition of controls. e, Ascertainment of exposure. f, Same method of ascertainment for cases and controls. g, Non-response rate.

**Appendix 2:** Forest plots of the insignificant results

**
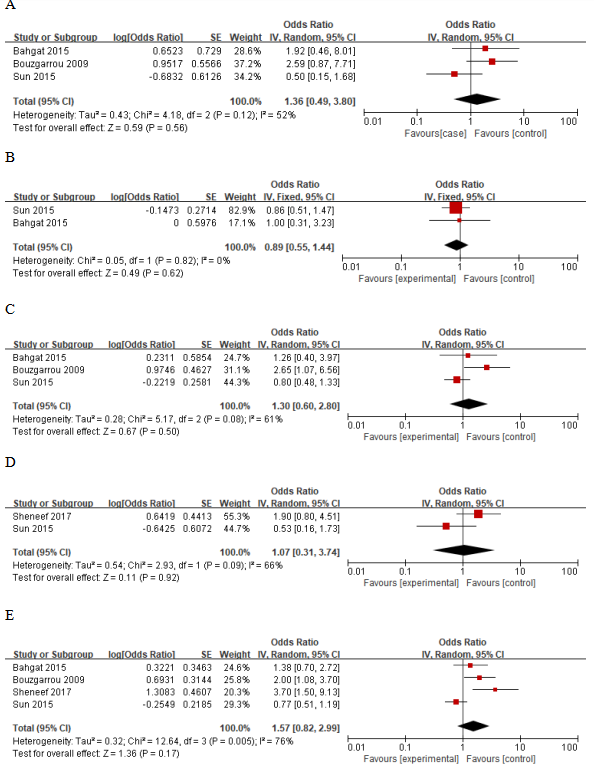
**

Figure 1. A, IFN-γ +874 TT vs AA; B, TA vs AA; C, TT+TA vs AA; D, TT vs TA+AA; E, T vs A.

**
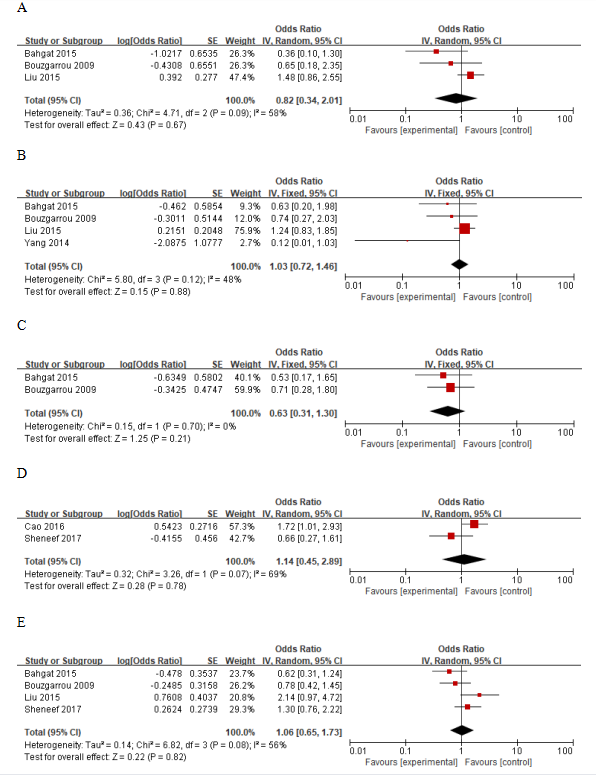
**

Figure 2. A, IL-10 -1082 GG vs AA; B, GA vs AA; C, GG+GA vs AA; D, AA vs GG+GA; E, G vs A.

**
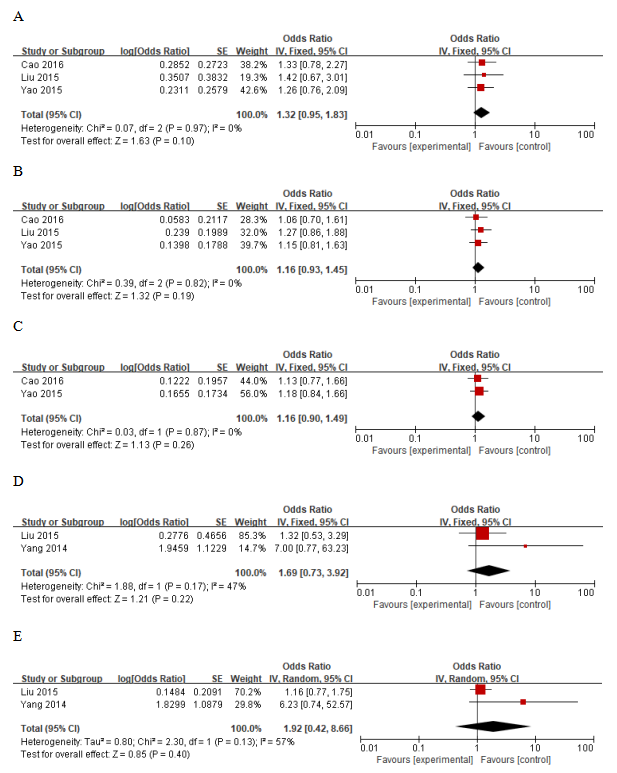
**

Figure 3. A, IL-10 -592 CC vs AA; B, AC vs AA; C, AC+CC vs AA; D, -819(rs1800871) TT vs CC; E, CT vs CC.

**
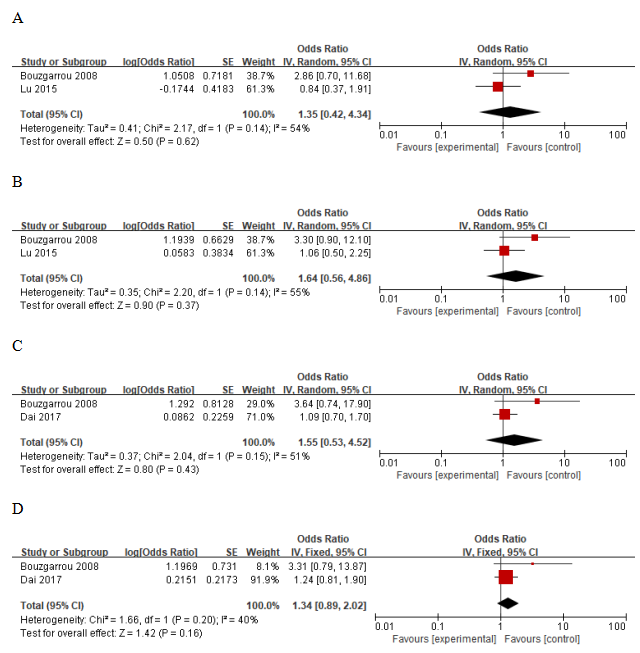
**

Figure 4. A, IL-18 -607 CC vs AA; B, CA vs AA; C, IL-18 -137(rs187238) GC vs CC; D, CG+GG vs CC.

**
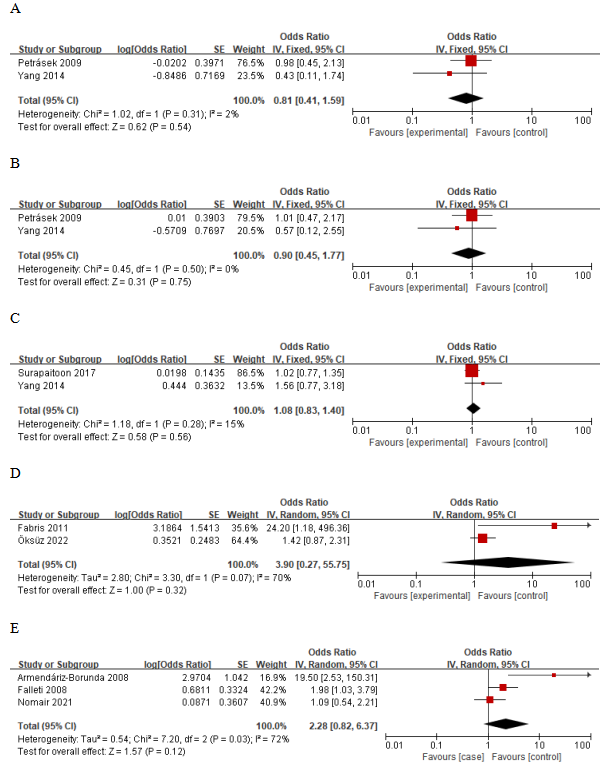
**

Figure 5. A, IL-1β -511 CC vs TT; B, CT vs TT; C, T vs C; D, IL-28 rs12979860 TT vs CT+CC; E, TGF-β1 Arg25Pro G vs C.

**
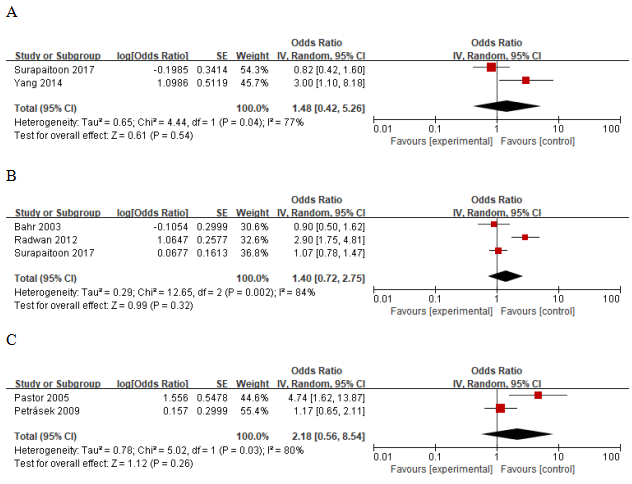
**

Figure 6. A, TNF-α -308 GA vs GG; B, A vs G; C, -238 GA vs GG+AA.

**Appendix 3:** Forest plots of subgroup analyses by different etiologies of liver cirrhosis

**
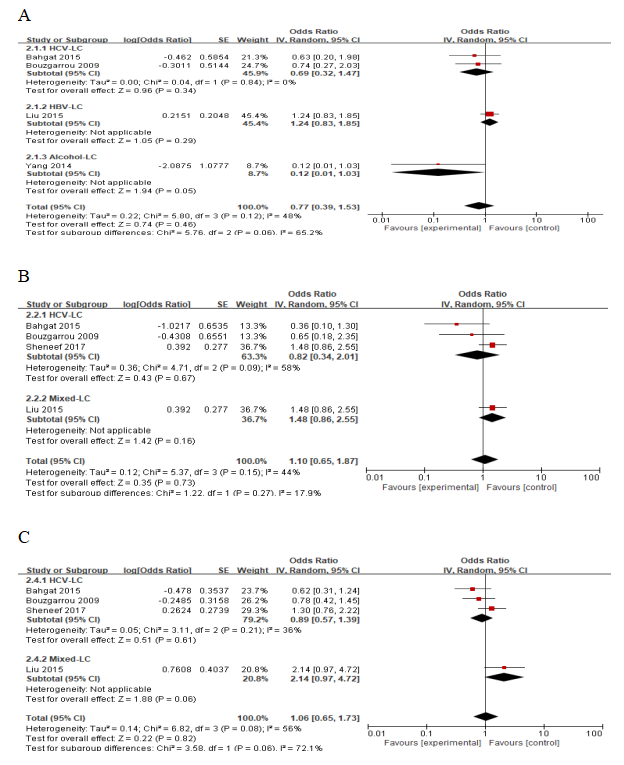
**

Figure 1. A, IL-10 -1082 GA vs AA; B, GG vs AA; C, G vs A.

**
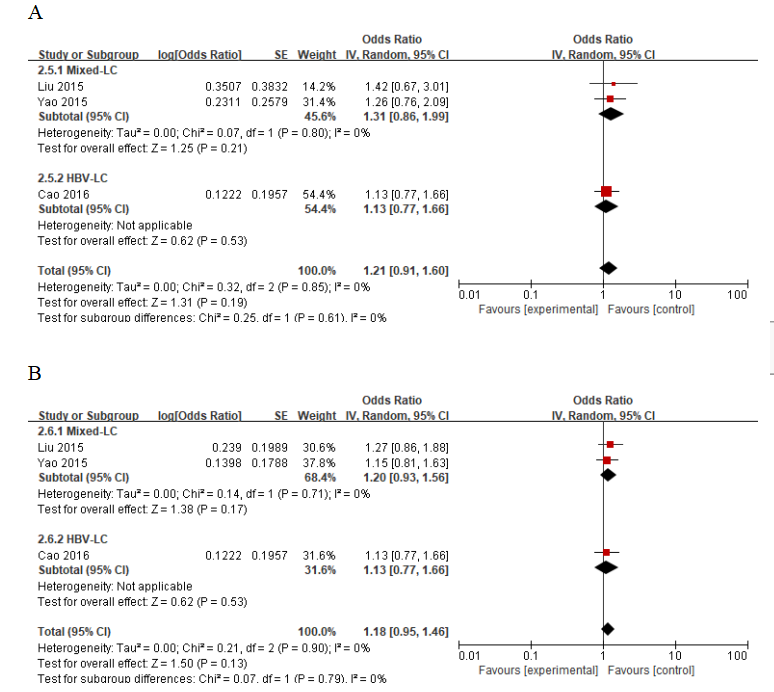
**

Figure 2. A, IL-10 -592 CC vs AA; B, AC vs AA.**
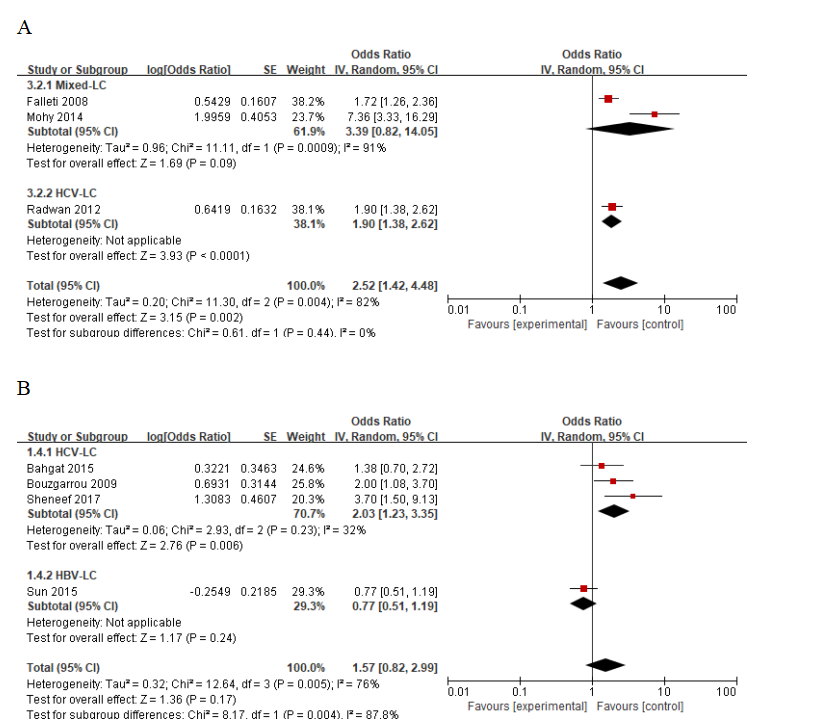
**

Figure 3. A, TGF-β1 Arg25Pro G vs C; B, TNF-α -308 A vs G.

**
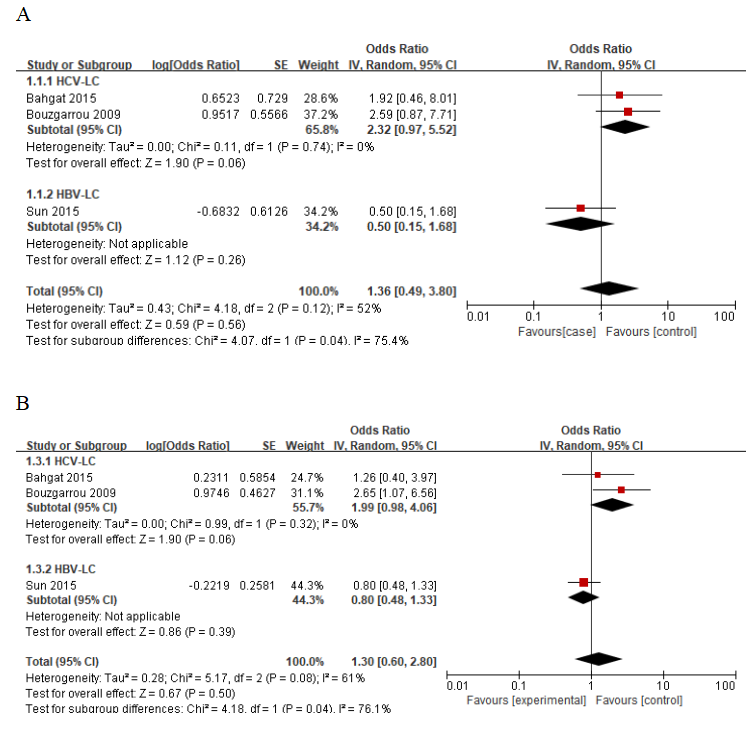
**

Figure 4. A, IFN-γ +874 TT vs AA; B, TT+TA vs AA.

**Appendix 4:**  Association between gene polymorphisms of other inflammatory factors and liver cirrhosis

|  | Study | Case | | Control | | OR (95% CI) |
| --- | --- | --- | --- | --- | --- | --- |
|  |  | Type | No. | Type | No. |  |
| CXCL-1 rs4074G/A | Nischalke 2013[1] | Alcohol-LC | 458 | HC | 342 |  |
| GA+AA vs GG |  |  |  |  |  | **1.62 (1.21, 2.15)** |
| IFN-γ +2109A/G | Sun 2015[2] | HBV-LC | 126 | HC | 173 |  |
| AG vs AA |  |  |  |  |  | 0.63 (0.38, 1.04) |
| GG vs AA |  |  |  |  |  | **0.32 (0.13, 0.79)** |
| G vs A |  |  |  |  |  | **0.57 (0.39, 0.83)** |
| AG+GG vs AA |  |  |  |  |  | **0.55 (0.34, 0.88)** |
| GG vs AA+AG |  |  |  |  |  | **0.39 (0.15, 0.93)** |
| IFR5 rs13242262A/T | Sy 2018[3] | HBV-LC | 131 | CHB | 99 |  |
| AT vs AA |  |  |  |  |  | 1.00 (0.5, 2.00) |
| TT vs AA |  |  |  |  |  | **3.10 (1.20,7.80)** |
| T vs A |  |  |  |  |  | **1.50 (1.10, 2.30)** |
| AT+TT vs AA |  |  |  |  |  | 1.30 (0.70, 2.60) |
| TT vs AA+AT |  |  |  |  |  | **2.80 (1.30, 5.90)** |
| rs10488630A/G |  |  |  |  |  |  |
| AG vs AA |  |  |  |  |  | 1.60 (0.90, 2.90) |
| GG vs AA |  |  |  |  |  | 3.00 (1.00, 9.00) |
| G vs A |  |  |  |  |  | **1.70 (1.10, 2.60)** |
| AG+GG vs AA |  |  |  |  |  | 1.80 (1.00, 3.20) |
| GG vs AA+AG |  |  |  |  |  | 2.40 (0.80, 7.20) |
| IL-13 rs1800925A/T | Long 2015[4] | Schistosoma-LC | 339 | HC | 307 |  |
| T vs A |  |  |  |  |  | **1.49 (1.03, 2.13)** |
| rs20541A/T |  |  |  |  |  |  |
| A vs T |  |  |  |  |  | 0.91 (0.67, 1.24) |
| IL-17 rs4711998A/G | Wang 2016[5] | Mixed-LC | 132 | HC | 171 |  |
| G vs A |  |  |  |  |  | **1.54 (1.06, 2.25)** |
| AG vs AA |  |  |  |  |  | **1.76 (1.10, 2.82)** |
| GG vs AA |  |  |  |  |  | 2.01 (0.59, 6.84) |
| AG+GG vs AA |  |  |  |  |  | **1.78 (1.12, 2.82)** |
| GG vs AA |  |  |  |  |  | 1.58 (0.47, 5.30) |
| rs2275913A/G |  |  |  |  |  |  |
| G vs A |  |  |  |  |  | 1.21 (0.88, 1.67) |
| AG vs AA |  |  |  |  |  | 1.60 (0.90, 2.83) |
| GG vs AA |  |  |  |  |  | 1.53 (0.76, 3.05) |
| AG+GG vs AA |  |  |  |  |  | 1.58 (0.91, 2.73) |
| GG vs AA |  |  |  |  |  | 1.09 (0.63, 1.91) |
| rs763780C/T |  |  |  |  |  |  |
| C vs T |  |  |  |  |  | 1.19 (0.81, 1.76) |
| CT vs TT |  |  |  |  |  | 0.94 (0.59, 1.54) |
| CC vs TT |  |  |  |  |  | 3.54 (0.91, 3.79) |
| CT+CC vs TT |  |  |  |  |  | 1.07 (0.67, 1.70) |
| CC vs TT+CT |  |  |  |  |  | 3.61 (0.94, 13.90) |
| IL-1α -889 | Bahr 2003[6] | HCV-LC | 52 | HC | 200 |  |
| A2A2 |  |  |  |  |  | 0.60 (0.20, 2.20) |
| A2 |  |  |  |  |  | 1.00 (0.60, 1.60) |
| rs3783553 | Du 2014[7] | HBV-LC | 358 | ASC+CHB | 632 |  |
| del\ins |  |  |  |  |  | 0.91 (0.79, 1.05) |
| ins\ins |  |  |  |  |  | 1.42 (0.91, 2.19) |
| ins/ins+del/ins |  |  |  |  |  | 0.93 (0.71, 1.22) |
| ins/ins |  |  |  |  |  | **1.54 (1.03, 2.33)** |
| IL-1β -3953 | Bahr 2003[6] | HCV-LC | 52 | HC | 200 |  |
| A2A2 |  |  |  |  |  | 1.10 (0.30, 3.90) |
| A2 |  |  |  |  |  | 1.10 (0.60, 1.80) |
| -31C/T | Petrásek 2009[8] | Alcohol-LC | 100 | HC | 180 |  |
| CT vs CC |  |  |  |  |  | 1.01 (0.47, 2.19) |
| TT vs CC |  |  |  |  |  | 0.98 (0.45, 2.16) |
| IL-2 -330T/G | Marcos 2008[9] | Alcohol-LC | 96 | HC | 161 |  |
| TG vs TT |  |  |  |  |  | 1.14 (0.68, 1.92) |
| GG vs TT |  |  |  |  |  | 0.29 (0.81, 1.04) |
| TG+GG vs TT |  |  |  |  |  | 0.96 (0.58, 1.60) |
| GG vs TT+TG |  |  |  |  |  | **0.27 (0.08, 0.96)** |
| +114T/G | Peng 2014[10] | HBV-LC | 67 | HC | 105 |  |
| TG vs TT |  |  |  |  |  | 0.90 (0.42, 1.92) |
| GG vs TT |  |  |  |  |  | 1.92 (0.88, 4.19) |
| G vs T |  |  |  |  |  | 1.49 (0.97, 2.31) |
| -384T/G |  |  |  |  |  |  |
| TG vs TT |  |  |  |  |  | 0.62 (0.31, 1.28) |
| GG vs TT |  |  |  |  |  | 1.92 (0.52, 4.30) |
| G vs T |  |  |  |  |  | 1.47 (0.92, 2.36) |
| IL-22 rs1026788A/G | Gao 2019[11] | HBV-LC | 264 | CHB | 103 |  |
| AG vs AA |  |  |  |  |  | 1.13 (0.67, 1.91) |
| GG vs AA |  |  |  |  |  | **2.34 (1.11, 4.97)** |
| G vs A |  |  |  |  |  | **1.41 (1.02, 1.97)** |
| rs1179249A/C |  |  |  |  |  |  |
| AC vs CC |  |  |  |  |  | 0.64 (0.39, 1.04) |
| AA vs CC |  |  |  |  |  | **0.38 (0.17, 0.84)** |
| A vs C |  |  |  |  |  | **0.65 (0.46, 0.91)** |
| rs2046068A/C |  |  |  |  |  |  |
| AC vs AA |  |  |  |  |  | 1.16 (0.73, 1.88) |
| CC vs AA |  |  |  |  |  | 1.13 (0.35, 3.67) |
| C vs A |  |  |  |  |  | 1.11 (0.75, 1.65) |
| rs2227472A/G |  |  |  |  |  |  |
| AG vs AA |  |  |  |  |  | 1.24 (0.74, 2.07) |
| GG vs AA |  |  |  |  |  | **2.57 (1.23, 5.4)** |
| G vs A |  |  |  |  |  | **1.49 (1.07, 2.07)** |
| rs2227473A/G |  |  |  |  |  |  |
| AG vs AA |  |  |  |  |  | 0.58 (0.06, 5.17) |
| GG vs AA |  |  |  |  |  | 0.48 (0.06, 4.17) |
| G vs A |  |  |  |  |  | 0.81 (0.50, 1.30) |
| rs2227485C/T |  |  |  |  |  |  |
| CT vs CC |  |  |  |  |  | 1.05 (0.62, 1.76) |
| TT vs CC |  |  |  |  |  | **2.21 (1.07, 4.59)** |
| T vs C |  |  |  |  |  | 1.38 (0.99, 1.91) |
| rs2227491A/G |  |  |  |  |  |  |
| AG vs AA |  |  |  |  |  | 1.10 (0.65, 1.85) |
| GG vs AA |  |  |  |  |  | **2.39 (1.13, 5.05)** |
| G vs A |  |  |  |  |  | **1.41 (1.01, 1.95)** |
| rs7314777T/C |  |  |  |  |  |  |
| TC vs TT |  |  |  |  |  | 1.20 (0.71, 2.05) |
| CC vs TT |  |  |  |  |  | 1.65 (0.18, 15.00) |
| C vs T |  |  |  |  |  | 1.21 (0.75, 1.94) |
| IL-27 -964A/G | Peng 2013[12] | HBV-LC | 65 | HC | 105 |  |
| AG vs AA |  |  |  |  |  | 0.74 (0.37, 1.46) |
| GG vs AA |  |  |  |  |  | 0.70 (0.29, 1.74) |
| G vs A |  |  |  |  |  | 0.80 (0.51, 1.26) |
| 2905T/G |  |  |  |  |  |  |
| TG vs TT |  |  |  |  |  | 1.03 (0.51, 2.08) |
| GG vs TT |  |  |  |  |  | 0.31 (0.04, 2.79) |
| G vs T |  |  |  |  |  | 0.83 (0.46, 1.52) |
| IL-28 rs8099917T/G | Jiao 2011[13] | HBV-LC | 100 | HC | 144 |  |
| TG+GG vs TT |  |  |  |  |  | 1.35 (0.55, 3.32) |
| G vs T |  |  |  |  |  | 1.46 (0.63, 3.40) |
| IL-33 rs1048274A/G | Ma 2021[14] | HBV-LC | 680 | HC | 840 |  |
| AG vs AA |  |  |  |  |  | 1.21 (0.95, 1.54) |
| GG vs AA |  |  |  |  |  | **1.66 (1.23, 2.23)** |
| AA vs AG+GG |  |  |  |  |  | **1.32 (1.05, 1.67)** |
| AA+AG vs GG |  |  |  |  |  | **1.46 (1.14, 1.87)** |
| A vs G |  |  |  |  |  | **1.27 (1.10, 1.46)** |
| rs10975519T/C |  |  |  |  |  |  |
| TC vs TT |  |  |  |  |  | 1.20 (0.94, 1.53) |
| CC vs TT |  |  |  |  |  | **1.75 (1.30, 2.36)** |
| TT vs TC+CC |  |  |  |  |  | **1.34 (1.06, 1.68)** |
| TT+TC vs CC |  |  |  |  |  | **1.55 (1.21, 2.00)** |
| T vs C |  |  |  |  |  | **1.30 (1.12, 1.50)** |
| rs4742170T/C |  |  |  |  |  |  |
| TC vs TT |  |  |  |  |  | 1.16 (0.92, 1.45) |
| CC vs TT |  |  |  |  |  | **1.84 (1.35, 2.51)** |
| TT vs TC+CC |  |  |  |  |  | **1.30 (1.05, 1.61)** |
| TT+TC vs CC |  |  |  |  |  | **1.69 (1.28, 2.23)** |
| T vs C |  |  |  |  |  | **1.31 (1.13, 1.52)** |
| IL-4 -589C/T | Lu 2014[15] | HBV-LC | 52 | HC | 147 |  |
| CT vs TT |  |  |  |  |  | 1.92 (0.92, 4.01) |
| CC vs TT |  |  |  |  |  | 2.83 (0.42, 18.88) |
| CT+CC vs TT |  |  |  |  |  | 1.98 (0.99, 4.08) |
| CC vs TT+CT |  |  |  |  |  | 2.23 (0.35, 14.43) |
| C vs T |  |  |  |  |  | 1.76 (0.97, 3.18) |
| -33C/T |  |  |  |  |  |  |
| CT vs TT |  |  |  |  |  | 1.84 (0.89, 3.82) |
| CC vs TT |  |  |  |  |  | 2.76 (0.51, 14.96) |
| CT+CC vs TT |  |  |  |  |  | 1.92 (0.95, 3.89) |
| CC vs TT+CT |  |  |  |  |  | 2.22 (0.43, 11.54) |
| C vs T |  |  |  |  |  | 1.73 (0.97, 3.07) |
| IL-6 rs1474347A/C | Abd El-Baky 2020[16] | HCV-LC | 22 | CHC | 22 |  |
| AA vs AC+CC |  |  |  |  |  | 0.27 (0.06, 1.23) |
| AC vs AA+CC |  |  |  |  |  | **5.70 (1.05, 31.07)** |
| rs10499563T/C | Xia 2020[17] | HBV-LC | 190 | HC | 246 |  |
| TC vs TT |  |  |  |  |  | 0.70 (0.46, 1.07) |
| CC vs TT |  |  |  |  |  | 0.38 (0.12, 1.19) |
| TC+CC vs TT |  |  |  |  |  | **0.66 (0.44, 0.99)** |
| C vs T |  |  |  |  |  | **0.67 (0.47, 0.95)** |
| rs2069837A/G |  |  |  |  |  |  |
| AG vs AA |  |  |  |  |  | **0.64 (0.43, 0.97)** |
| GG vs AA |  |  |  |  |  | **0.23 (0.07, 0.98)** |
| AG+GG vs AA |  |  |  |  |  | **0.60 (0.40, 0.90)** |
| G vs A |  |  |  |  |  | **0.62 (0.44, 0.87)** |
| -572G/C | Yang 2014[18] | Alcoho-LC | 40 | HC | 64 |  |
| GC vs GG |  |  |  |  |  | 1.02 (0.15, 6.88) |
| CC vs GG |  |  |  |  |  | 0.89 (0.14, 5.69) |
| G vs C |  |  |  |  |  | 1.11 (0.54, 2.27) |
| -174G/C | Surapaitoon 2017[19] | O viverrini-LC | 200 | HC | 200 |  |
| GC vs GG |  |  |  |  |  | 0.79 (0.50, 1.25) |
| CC vs GG |  |  |  |  |  | 0.73 (0.34, 1.53) |
| C vs G |  |  |  |  |  | 0.81 (0.58, 1.13) |
| IL-8 -251A/T | Qin 2012[20] | HBV | 80 | HC | 150 |  |
| AA vs TT |  |  |  |  |  | **0.14 (0.02, 0.87)** |
| AT vs TT |  |  |  |  |  | 0.58 (0.17, 1.95) |
| A vs T |  |  |  |  |  | **0.48 (0.25, 0.92)** |
| +781C/T |  |  |  |  |  |  |
| CC vs TT |  |  |  |  |  | 0.44 (0.06, 3.09) |
| CT vs TT |  |  |  |  |  | 0.35 (0.06, 2.24) |
| C vs T |  |  |  |  |  | 1.54 (0.77, 3.08) |
| IRF3 -925A/G | Kim 2004[21] | HCV-LC | 56 | CHC | 83 |  |
| GG vs AG+AA |  |  |  |  |  | 1.20 (0.56, 2.58) |
| AG vs GG+AA |  |  |  |  |  | 0.51 (0.26, 1.01) |
| AA vs GG+AG |  |  |  |  |  | 2.19 (0.82, 5.85) |
| G vs A |  |  |  |  |  | 0.98 (0.60, 1.58) |
| A vs G |  |  |  |  |  | 1.03 (0.63, 1.67) |
| LT-α +252A/G | Surapaitoon 2017[19] | O viverrini-LC | 200 | HC | 200 |  |
| AG vs AA |  |  |  |  |  | 1.60 (0.95, 2.65) |
| GG vs AA |  |  |  |  |  | 1.16 (0.66, 2.01) |
| G vs A |  |  |  |  |  | 1.07 (0.80, 1.43) |
| MIF -173G/C | Zhang 2013[22] | HBV-LC | 73 | HC | 90 |  |
| CC vs GC+CC |  |  |  |  |  | **4.07 (1.06, 15.67)** |
| TGF-β1 -800G/A | Falleti 2008[23] | Mixed-LC | 188 | HC | 140 |  |
| G vs A |  |  |  |  |  | 1.51 (0.83, 2.73) |
| Leu10ProC/T |  |  |  |  |  |  |
| T vs C |  |  |  |  |  | 1.21 (0.89, 1.65) |
| TNFAIP3 rs148314165T | Li 2017[24] | HBV-LC | 158 | CHB | 165 |  |
| TT vs Tdel |  |  |  |  |  | 1.06 (0.56, 2.00) |
| T vs del |  |  |  |  |  | 1.05 (0.57, 1.94) |
| rs200820567T/A |  |  |  |  |  |  |
| TT vs TA+AA |  |  |  |  |  | 1.06 (0.56, 2.00) |
| T vs A |  |  |  |  |  | 1.05 (0.57, 1.94) |
| rs2230926G/T | Zhang 2015[25] | HBV-LC | 167 | CHB | 183 |  |
| TT |  |  |  |  |  | 1.25 (0.47, 3.31) |
| T vs G |  |  |  |  |  | 1.24 (0.47, 3.25) |
| TNF-α -857C/T | Qiu 2012[26] | HBV-LC | 196 | SR | 189 |  |
| CC vs CT+TT |  |  |  |  |  | **1.57 (1.04, 2.35)** |
| C vs T |  |  |  |  |  | **1.47 (1.06, 2.04)** |
| -863C/A |  |  |  |  |  |  |
| AA vs CC+CA |  |  |  |  |  | **3.83 (1.34, 10.96)** |
| C vs A |  |  |  |  |  | **1.81 (1.26, 2.61)** |
| VEGFA +936C/T | Giacalone 2011[27] | Mixed-LC | 79 | HC | 162 |  |
| CC vs CT+TT |  |  |  |  |  | 0.71 (0.39, 1.29) |
| CT vs CC+TT |  |  |  |  |  | 1.42 (0.77, 2.61) |
| TT vs CC+CT |  |  |  |  |  | 1.00 (0.19, 5.96) |
| C vs T |  |  |  |  |  | 1.30 (0.78, 2.20) |

Schistosoma, schistosoma infection; ASC, asymptomatic hepatitis; SR, spontaneously recovered.

**References**

1. Nischalke, H.D., et al., *Influence of the CXCL1 rs4074 A allele on alcohol induced cirrhosis and HCC in patients of European descent.* PLoS One, 2013. **8**(11): p. e80848.

2. Sun, Y., et al., *Interferon gamma polymorphisms and hepatitis B virus-related liver cirrhosis risk in a Chinese population.* Cancer Cell Int, 2015. **15**: p. 35.

3. Sy, B.T., et al., *Genetic variants of interferon regulatory factor 5 associated with chronic hepatitis B infection.* World J Gastroenterol, 2018. **24**(2): p. 248-256.

4. Long, X., et al., *An IL-13 promoter polymorphism associated with liver fibrosis in patients with Schistosoma japonicum.* PLoS One, 2015. **10**(8): p. e0135360.

5. Wang, J., et al., *Association of IL-17A and IL-17F gene polymorphisms with chronic hepatitis B and hepatitis B virus-related liver cirrhosis in a Chinese population: A case-control study.* Clin Res Hepatol Gastroenterol, 2016. **40**(3): p. 288-296.

6. Bahr, M.J., et al., *Cytokine gene polymorphisms and the susceptibility to liver cirrhosis in patients with chronic hepatitis C.* Liver Int, 2003. **23**(6): p. 420-5.

7. Du, Y., et al., *Association of miRNA-122-binding site polymorphism at the interleukin-1 α gene and its interaction with hepatitis B virus mutations with hepatocellular carcinoma risk.* Front Med, 2014. **8**(2): p. 217-26.

8. Petrásek, J., et al., *Do common genetic variants in endotoxin signaling pathway contribute to predisposition to alcoholic liver cirrhosis?* Clin Chem Lab Med, 2009. **47**(4): p. 398-404.

9. Marcos, M., et al., *A new genetic variant involved in genetic susceptibility to alcoholic liver cirrhosis: -330T>G polymorphism of the interleukin-2 gene.* Eur J Gastroenterol Hepatol, 2008. **20**(9): p. 855-9.

10. Peng, Q., et al., *Association of IL-2 polymorphisms and IL-2 serum levels with susceptibility to HBV-related hepatocellular carcinoma in a Chinese Zhuang population.* Infect Genet Evol, 2014. **27**: p. 375-81.

11. Gao, Y.H., et al., *The role of IL22 polymorphisms on liver cirrhosis in patients with hepatitis B virus: A case control study.* Medicine (Baltimore), 2019. **98**(44): p. e17867.

12. Peng, Q., et al., *Association of IL27 gene polymorphisms and HBV-related hepatocellular carcinoma risk in a Chinese population.* Infect Genet Evol, 2013. **16**: p. 1-4.

13. Jiao, X.L., et al., *[Studies on the relationship between polymorphism of IL-28B rs8099917 and the outcome of HBV infection].* Zhonghua Liu Xing Bing Xue Za Zhi, 2011. **32**(11): p. 1143-7.

14. Ma, N., et al., *Genetic variants in IL33 and IL1RL1 genes confer susceptibility to HBV-related liver cirrhosis in Chinese Han population.* Infect Genet Evol, 2021. **94**: p. 104983.

15. Lu, Y., et al., *Role of IL-4 gene polymorphisms in HBV-related hepatocellular carcinoma in a Chinese population.* PLoS One, 2014. **9**(10): p. e110061.

16. Abd El-Baky, R.M., et al., *Impact of interleukin IL-6 rs-1474347 and IL-10 rs-1800896 genetic polymorphisms on the susceptibility of HCV-infected Egyptian patients to hepatocellular carcinoma.* Immunol Res, 2020. **68**(3): p. 118-125.

17. Xia, C., et al., *Genetic polymorphisms of interleukin-6 influence the development of hepatitis B virus-related liver cirrhosis in the Han Chinese population.* Infect Genet Evol, 2020. **84**: p. 104331.

18. Yang, A.M., et al., *Interleukin 10 promoter haplotype is associated with alcoholic liver cirrhosis in Taiwanese patients.* Kaohsiung J Med Sci, 2014. **30**(6): p. 291-8.

19. Surapaitoon, A., et al., *Subsets of Inflammatory Cytokine Gene Polymorphisms are Associated with Risk of Carcinogenic Liver Fluke Opisthorchis viverrini-Associated Advanced Periductal Fibrosis and Cholangiocarcinoma.* Korean Journal of Parasitology, 2017. **55**(3): p. 295-304.

20. Qin, X., et al., *The IL-8 gene polymorphisms and the risk of the hepatitis B virus/infected patients.* DNA Cell Biol, 2012. **31**(6): p. 1125-30.

21. Kim, M.S., et al., *Genetic polymorphisms of alcohol-metabolizing enzymes and cytokines in patients with alcohol induced pancreatitis and alcoholic liver cirrhosis.* The Korean journal of gastroenterology = Taehan Sohwagi Hakhoe chi, 2004. **43**(6): p. 355-363.

22. Zhang, K., et al., *Relationship between MIF-173 G/C polymorphism and susceptibility to chronic hepatitis B and HBV-induced liver cirrhosis.* Cellular Immunology, 2013. **281**(2): p. 113-116.

23. Falleti, E., et al., *TGF-beta1 genotypes in cirrhosis: relationship with the occurrence of liver cancer.* Cytokine, 2008. **44**(2): p. 256-61.

24. Li, N., et al., *Association of the tandem polymorphisms (rs148314165, rs200820567) in TNFAIP3 with chronic hepatitis B virus infection in Chinese Han population.* Virol J, 2017. **14**(1): p. 148.

25. Zhang, P., et al., *Association between TNFAIP3 nonsynonymous single-nucleotide polymorphism rs2230926 and chronic hepatitis B virus infection in a Chinese Han population.* Virol J, 2015. **12**: p. 33.

26. Qiu, B., et al., *Association of TNF-α promoter polymorphisms with the outcome of persistent HBV infection in a northeast Chinese Han population.* Acta Biochim Biophys Sin (Shanghai), 2012. **44**(8): p. 712-8.

27. Giacalone, A., et al., *Association between single nucleotide polymorphisms in the cyclooxygenase-2, tumor necrosis factor-α, and vascular endothelial growth factor-A genes, and susceptibility to hepatocellular carcinoma.* Omics, 2011. **15**(3): p. 193-6.
